# Professional identity and experience of undergraduate students: an analysis of semantic networks

**DOI:** 10.1186/s41155-021-00179-8

**Published:** 2021-05-26

**Authors:** Luara Carvalho, Elisa Maria Barbosa de Amorim-Ribeiro, Marcelo do Vale Cunha, Luciana Mourão

**Affiliations:** 1 Universidade Salgado de Oliveira, Marechal Deodoro, 217-Bloco A, Niterói, Rio de Janeiro 24030-060 Brazil; 2 Instituto Federal da Bahia, Gileno de Sá Oliveira, 271, Recanto dos Pássaros, Barreiras, Bahia 47808-006 Brazil

**Keywords:** Professional identity, Semantic network, Network analysis, Professional experience, Undergraduate students

## Abstract

Work experiences during undergraduate studies can be remarkable in the journey of undergraduate students. The objective of this study was to assess, by analyzing semantic networks, the role of work experiences in the meanings those individuals attribute to professional identity. The sample consisted of 2291 students (60% women) divided into three groups: do not work, work in a field related to their course, work in a field not related to their course. The semantic networks of these groups were composed of words uttered from the professional identity prime. We chose to work with the critical network, obtained from the analysis of the incidence-fidelity indexes of the word pairs. The results evidence that work experiences are related to how undergraduate students attribute meaning to professional identity, in such a way that three different networks were formed for these groups. The network of those who work outside their field was the only one that integrated words with negative content, while the semantic networks of those who do not work and those who work in their field, despite containing words that do not always coincide, present a similar macrostructure. We conclude that work experiences play an important role in the meanings that undergraduate students attribute to professional identity. The study innovates by revealing elements of professional-identity construction, besides allowing for reflections on the effects of work experiences during the college period.

## Introduction

Professional identity is conceptualized as the perception of inclusion in a certain social group that shares a specific domain of technical and work-related knowledge (Gondim, Bendassolli, & Peixoto, 2016; Tajfel, 1983). It is also defined as a cognitive and dynamic process that occurs through narratives actively constructed through social interaction (Guichard, 2009; Savickas, 2013). Thus, professional identity is in a constant construction process and, during undergraduate studies, this process receives greater emphasis due to the various stimuli to which students are exposed. For instance, it is common to have questions, such as: Do I feel close to my future colleagues in terms of worldview, attitudes, tastes, and interests? What types of work environments would suit me? Which subfields of my profession do I identify with most? (Guichard, 2018).

In higher education, three aspects can be listed for the construction of professional identity. Firstly, by recognizing themselves as professionals, the students begin to develop knowledge, skills, attitudes, and values that are similar to those of other members of the profession. Secondly, in doing so, they become different from those who are not part of a certain field. And thirdly, they identify themselves as members of the category of people in that profession (Trede, Macklin, & Bridges, 2012).

Thus, undergraduate students are always in the tension between identification with a certain group that shares the same theoretical and practical domain and opposition to or distancing from other professional groups (Gondim et al., 2016). In the wake of studies on professional identity in higher education, systematic literature reviews suggest a research agenda on the meaning of the construct from the perspective of this target audience (Trede et al., 2012; Vozniak, Mesquita, & Batista, 2016).

In this context, the development of professional identity is understood as a dynamic process dependent on self-perception and interpersonal and cultural relations, therefore presenting a congruence with the Archway Model proposed by Super (1980, 1990), which summarizes career development and its influences by biological, psychological, and socioeconomic determinants. This analogy is represented literally by an arch, which considers different roles and life stages. Its support base is divided into two dimensions: (*i*) biographical dimension, which is about the importance of personality for career choices and synthesized by accomplishments, and (*ii*) geographic dimension, which reflects the influence that social policy has on career development through practices at work.

The model also encompasses the stages of development established by Super (1990), in which the left pillar of the arch represents the personal-biographical dimension, such as needs, values, interests, intelligence, and special skills, and is associated with childhood and adolescence. The right pillar of the arch, in turn, represents the social-geographic dimension, such as economic resources, economy, job market, family, school, community, and peer groups, and is related to adulthood and maturity. Each stage requires tasks that vary by age and social expectations, which will be performed in different roles.

The two pillars of the arch interact mutually through individual and social aspects, having the self as a link (Super, 1990). At this junction, professional identity is constructed, which is composed of people’s view of themselves and their identification with environmental aspects. In this sense, professional identity has a social component, as people do not construct this identity with subjective elements only, but also with the various social and contextual inputs they receive. In this logic, the self would be responsible for establishing the self-concept in professional terms, which is constituted in the subjective perspective and evokes the meanings of each person’s values, interests, and capabilities (Alfonso et al., 2020; Guichard, 2009; Oliveira, Melo-Silva, Taveira, & Postigo, 2019).

Therefore, it is the self that allows us to understand that people would have different images of their professional identity, even if they are in the same environment and exposed to the same types of stimulus. In the case of undergraduate students, professional identity is under construction. It arises from the interaction between an individual’s skills and social learning, the latter deriving from experiences developed during undergraduate studies and possible work experiences.

Different studies point to the effects of practical experiences inside and beyond higher education institutions (HEIs) for the construction of professional identity and evidence work experiences in one’s field of training (Hunter, Laursen, & Seymour, 2007; Trede et al., 2012). Meanwhile, learning opportunities that take place outside classrooms bring benefits to students (Mourão, Carvalho, & Monteiro, 2020).

This view of learning from experience is anchored in Kolb’s Theory of Experiential Learning (1984), which considers a cyclical process comprising four modes of learning: by thinking (conceptualization), by doing (active experimentation), by experimenting (concrete experience), and by reflecting on what has been seen and heard (reflective observation). Applying that theory to the construction of professional identity among undergraduate students, it would be assumed that this process of acquisition of knowledge, attitudes, and competencies would be continuous throughout training (Mourão et al., 2020). Having the possibility of articulating theory with practice allows these individuals to identify themselves more as professionals than as students and increases their identification with a certain workgroup (Oliveira et al., 2019).

Thus, there is a greater gain for students when the experiences in the workplace have a connection between what is taught at the university and the work activities carried out (Hunter et al., 2007). The contribution of work experience within the field of the course to the development of professional identity may be even greater than the contribution given by the university itself (West & Chur-Hansen, 2004). On the one hand, the intersection between university and work is integrated into the individual and relational perspectives of work-related learning (Weerdt, Bouwen, Corthouts, & Martens, 2006). On the other hand, it is worth questioning whether work experiences related to self-support and beyond one’s field of training would contribute to the construction of professional identity.

Work experiences during college, without a direct connection with one’s field of training, are approached in the literature from two perspectives. The first is that of gain of general competencies resulting from experiences in the world of work (Mourão et al., 2020). The second perspective is that the time spent on work activities competes with the dedication to higher education, making the training process more exhausting and reducing an individual’s possibility of taking advantage of opportunities provided by their undergraduate course (Fior & Mercuri, 2018; Silva & Teixeira, 2013). In this vein, even when it comes to working in one’s field of training, the time spent on work activities may compromise the quality of college training (Maier & Mattos, 2016).

Thus, studies in the area discuss whether work experience during the undergraduate period contributes to or harms students’ development. On the one hand, they can facilitate the development of practical skills and the transition to the labor market. On the other hand, work is not always related to experiences that allow the students to initiate contact with their area of work. Many students work out of the need to guarantee their livelihood and need to divide their time between studies and working hours, which can compromise their school performance (Maier & Mattos, 2016).

Considering this discussion of work experiences in the literature, we decided to research professional identity in three groups of students: undergraduate students who do not work, undergraduate students working in a field related to their course, undergraduate students working in a field not related to their course. These groups were chosen because the experiences in a field related to one’s training contribute to the construction of professional identity (Gondim et al., 2016; Silva & Teixeira, 2013).

A means to assess whether undergraduate students’ work experiences affect the meanings attributed to professional identity is by analyzing semantic networks. This analysis is inserted in the scientific paradigm of social-network analysis and allows assessing how meanings are constructed through patterns of connection between words (Pereira, Fadigas, Monteiro, Cordeiro, & Moret, 2016). Based on the mathematical theory of graphs, semantic networks have contributed to studies on cognitive processes, by making it possible to identify patterns of meaning in complex phenomena (Freeman, 2011).

The assumption of this type of analysis in the attribution of meanings regarding professional identity is that language provides the necessary words for people’s reflective processes concerning this study object. Thus, we work with the hypothesis that the concrete experiences of undergraduate students, whether enabled by personal or contextual pillars, are inputs for the co-construction of the self, which also spans the interpersonal relationships that attribute meaning to experiences (Froidevaux, 2018).

A reflection on professional identity leads us to think about our actions, who we want to be and what work we want to do, from the perspective that professional identity is socially built, culturally shaped, and linguistically narrated (Guichard, 2016). In this study, we used, within the framework of semantic networks, the analysis of critical networks, which highlights only the most relevant associations of the research phenomenon, with possibilities for reflection on cognitive-behavioral differences between individuals (Lima-Neto, Cunha, & Pereira, 2018).

The network analysis comes from an interdisciplinary field that studies the relationships between actors, forces, directions, and the contents of these relationships, as well as the macrostructure such interactions generate. Its peculiarity and richness are in prioritizing the relationship between actors or vertices as an analytical unit of a phenomenon (Borgatti & Lopez-Kidwell, 2011; Freeman, 2011), considering an analysis of clique networks. A clique is a set of mutually connected n vertices, in such a way that the basic element of a clique network is not the vertex but the clique (Fadigas & Pereira, 2013; Grilo et al., 2017).

A semantic clique network is a system to represent the knowledge established by a specific context and imbued with intention of functionality, in which vertices are words or concepts with meaning, the smallest unit of meaning is the sentence (for instance, a set of words uttered by an individual), and edges connect two words belonging to the same sentence (Cunha, Santos, Moret, & Pereira, 2020; Nascimento et al., 2018). Several authors have used this model, for instance, in oral discourse (Lima-Neto et al., 2018; Teixeira et al., 2010), in semantic networks of titles of scientific articles and course descriptions (Andrade, Barreto, Cunha, Ribeiro, & Pereira, 2019; Pereira et al., 2016), as well as in networks of uttered words (Lopes et al., 2015).

Thus, we adopt as an assumption the perception of language as a central mechanism to construct and constitute subjective social realities (Guichard, 2016), which can be interpreted from the analysis of semantic networks (Freeman, 2011). In the light of the foregoing, this study aims to assess, by analyzing semantic networks, the role of work experiences in the meanings that undergraduate students attribute to professional identity. The next section details the method defined for the study.

## Method

This exploratory study compares critical networks of three groups of undergraduate students to discuss the role of work experiences in the meanings that they attribute to professional identity. We chose to work with data collection by evocation and with the analysis of semantic networks according to the structures of meaning they offer based on the connections between words.

The method traditionally used for analysis with evocations is prototypical analysis, based on the paradigm of social representations, and which analyzes the evoked words based on the frequency and order of evocation (Abric, 1994). This analysis does not establish an association between the words that each participant evoked. In this sense, this method provides a list of words allocated in different quadrants, each with different meanings. Even when evaluating co-occurrences between words, they differ from the network in that they do not have discursive paths between words. In this sense, given the objective of this study, we opted for the use of social network analysis, based on semantic networks, to obtain discursive paths, which allow us to differentiate the meaning attributed to identical words, whose links to certain niches of words give rise to other interpretations.

Thus, semantic networks are characterized by structures of meaning formed by the connections between words. The group of words each participant evoked generates a click, that is, a group of mutually connected nodes. So, the main advantage of using semantic networks is the preservation of the meaning structure each participant attributed to the object of study (in the case of this article, professional identity). Therefore, the technique allows words (vertices) common to the cliques to connect to other cliques (meanings attributed by each participant), forming a macro sense structure of the object of study. The analysis holds tools that allow a refinement, in the sense of disregarding weak associations between words, through the incidence-fidelity (IF) index. In this sense, the analysis of semantic networks allowed us to obtain not only a structure of macro sense for professional identity, but also the most relevant network to understand these meanings, based on a progressive process of filtering words and cliques, culminating in the critical network.

Another advantage of semantic networks is that they permit analyzing the intermediation measure, which allows the identification of words that are necessary paths among other words. Thus, words with high intermediation add greater value to understand the axes of meaning related to the object studied, which does not occur when there is only the analysis of frequency and order of the evoked words.

### Participants

The sample consisted of 2291 undergraduate students (60% women) aged from 19 to 64 years old (*M* = 27; *SD* = 7.9) and mostly single (83%). The participants were enrolled in public (30%) and private (70%) HEIs located in the five Brazilian regions, namely, Southeast (80%), South and Northeast (14%), and North and Midwest (6%). The inclusion criterion was to be enrolled as of the second semester of the course, as those newly arrived at college are going through an adaptation period due to the significant change that occurs after entering higher education. The researched courses comprised the three groupings established in the Brazilian higher education, namely, Humanities (45%); Life Sciences (35%); and Exact, Technological and Multidisciplinary Sciences (20%). The sample included 51% of students attending until the first half of the course and 49% of students enrolled in the second half of the course.

In this study, semantic networks were generated for three groups, namely, (*i*) undergraduate students who do not work (*does not work* group), with 855 participants; (*ii*) undergraduate students working in a field related to their course (*works in the field* group), with 821 participants; (*iii*) undergraduate students working in a field not related to their course (*works outside the field* group), with 615 participants. The criterion used to determine the groups was through a self-reported measure, in which the participants answered the question about work experience in three alternative answers: (1) “I work in an area related to my undergraduate course”; (2) “I work in an area unrelated to my undergraduate course”; (3) “I still don’t work”.

### Instruments

In this study, the semantic network was composed of words the undergraduate students uttered from the “professional identity” prime. Thus, the only question asked to the participants other than those concerning sociodemographic data was, “What words come to mind when you think about your professional identity?” Utterance was chosen because the words that come from this data collection method are less impacted by social desirability in a conscious field, being more involved with the research subjects’ interpretation (self) of professional identity.

As a stimulus, three answer lines were presented after the driving question for the respondents to include a set of three words to compose the semantic network. Also, the questionnaire included sociodemographic data (sex, age, marital status, region, type of HEI, course period, and field) and work-related questions (paid activity and practice field).

### Ethical and data collection procedures

This study received approval from a Research Ethics Committee and complied with all expected ethical principles, including the confidentiality of individual information and the right to voluntary participation and withdrawal from the research at any time. All participants authorized the inclusion of their data by signing the free and informed consent form, and the collection was carried out online and in person. For the online collection, an invitation was sent by email and through other social networks, based on the disclosure of those who had already completed the survey, characterizing the use of the snowball technique. Data were collected in person as well, with the questionnaire printed, at five HEIs, which had previously authorized the application.

In the analysis of semantic networks, one of the main indicators is the IF index, which was proposed to investigate the semantic clique networks of oral discourses among undergraduate students from the Physics and Psychology courses (Teixeira et al., 2010). This index considers both the frequency of occurrence of the word pair (incidence) and the likelihood of the pair occurring in the context in which at least one word was uttered (fidelity). With the IF index acting as a filter, it is possible to identify an optimal network configuration, known as the critical network. Critical networks gain relevance for being a type of network that contains maximum representative information with minimum textual residue (Cunha et al., 2020). To obtain these networks, seven successive steps were followed.

At step 1, the uttered words were treated, in a way that verbs were converted to their infinitive form and the other ones to nouns. All diacritical marks were removed, and the phrases or sequences of words with meaning were grouped to form one single word (example, quality of life appeared as qualityoflife).

At step 2, the participants were divided into the three established groups (*does not work*, *works in the field*, and *works outside the field*), and for each group, a clique network was built (following the groupings of three words uttered by the participants). The networks were generated using the packages and dictionaries proposed by Teixeira et al. (2010). The packages perform three steps. The first refers to the grammatical classification of words, removing terms such as article, preposition, pronoun, and number. This step uses the UNITEX dictionary (https://unitexgramlab.org/language-resources) as a basis and was necessary, as some participants put more than one word in each of their three responses (e.g., be successful, earn my money). The second stage refers to the calculation of word frequencies, which makes it possible to determine the IF values of each pair of words that co-occur in a sentence. The third stage refers to the construction of the semantic network with its edges weighted by the IF.

In step 3, the inflection points as to the critical network were identified for each one of the groups, that is, the most significant associations, both in terms of frequency and likelihood of co-occurrence. This was possible through the IF index, which takes into account how often a word pair appears in the text (incidence) and how often at least one word from the pair is uttered (fidelity). After calculating the IF of each word pair, it was possible to identify a critical point responsible for a structural change in the network, that is, from that point, the removal of another word pair dissolves the network (Teixeira et al., 2010). Thus, to identify the main axes of the meaning attributed by each group to professional identity, a progressive filtering process was conducted through IF calculation (Cunha et al., 2020; Teixeira et al., 2010). All word pairs with IF below this limit value were removed from the semantic network, which allowed identifying the critical network of professional identity for each one of the three researched groups.

At step 4, the critical network was created by subtracting all words above the cutoff point set in the previous step. The network resulting from this process has a pattern that represents the discourse with maximum information and minimum textual residue. In this configuration, the critical network contains word pairs with a high association power, responsible for keeping the network connected (Cunha et al., 2020). In addition to being used as a criterion to reach the critical network of meaning, the IF was used as a strength parameter to measure the relationship between word pairs.

In step 5, calculations were made from the giant component of the critical network to identify the elements that make up the structure of the semantic networks of the three groups. The giant component of a network is the largest component of connected vertices, i.e., it is the component that contains more than half of the network’s vertices (Newman, 2003).

In step 6, the macrostructural data of the networks of each group were identified. Such data can be considered as the indexes of the network. They are number of vertices or words, represented by (*n*); number of edges (*m*); average degree (〈*k*〉), which corresponds to the average number of incident connections between the vertices in the network; density (Δ), equivalent to the proportion between existing edges and total possible edges; diameter (*D*), which is the greatest geodesic distance between two words; and, finally, average shortest path (*L*), which represents the shortest path between two words (Newman, 2003).

**Fig. 1:**
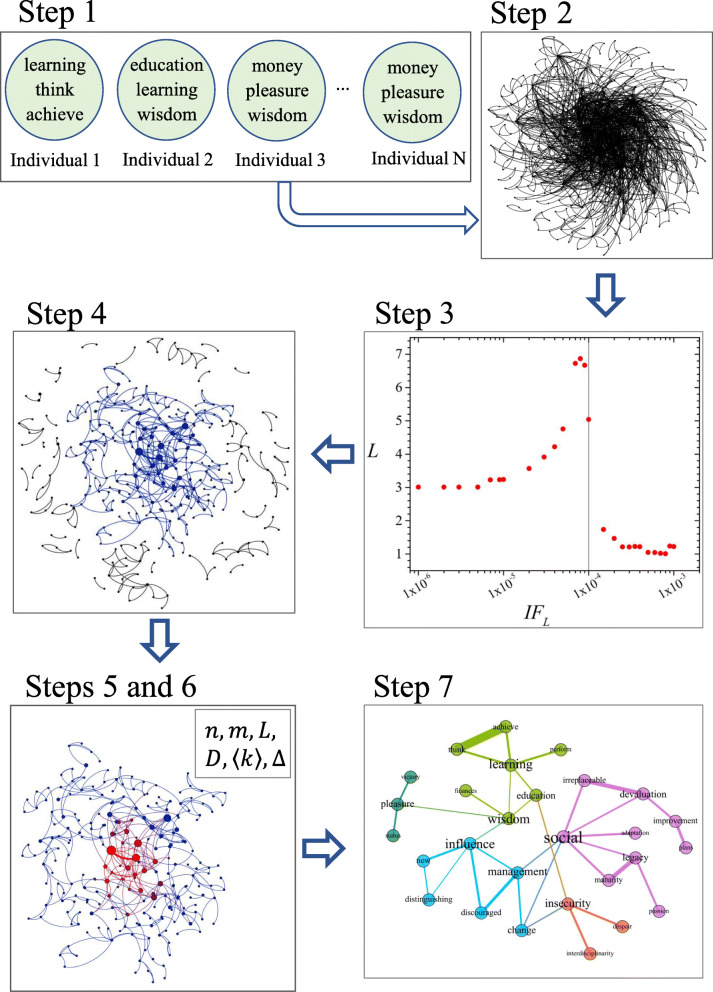
Summary of the seven steps of the data analysis procedures*. Notes.* step 1 words uttered by the individuals, and cliques formation; step 2 cliques network of uttered words; step 3 cutoff point for the critical network; step 4 critical network filtered; step 5 giant component of the critical network; step 6 macro-structural data of the network; step  7 subgroups of words in the largest community

Finally, in step 7, with the aid of the modularity algorithm, it was possible to identify subgroups of words more densely connected compared to the rest of the network (Blondel, Guillaume, Lambiotte, & Lefebvre, 2008; Lopes et al., 2015). The figures of the networks, in their turn, were prepared based on the largest community of the giant component of the critical network. This ascending filtering path of the network (critical network, giant component, and largest community of the critical network) allowed a comparative inspection of each group’s semantic networks, a key strategy to assess the role of work experiences in the formation of professional identity. Figure 1 summarizes the data analysis procedures adopted in the critical network analysis.

These analyses were performed with the aid of the Gephi software, version 0.9.2 (available at www.gephi.org). The results will be presented in the next section, considering the analysis of the critical networks of each one of the three researched groups (does not work, works in the field, and works outside the field), as well as a comparison of the macrostructural indicators of these groups’ critical networks.

## Results

Faced with the proposal of associating words with the professional identity term, the undergraduate students participating in the study uttered altogether 6694 observed words (a total of 870 different words), in accordance with each group’s work experiences: (1) *does not work* (2498—515 different words); (2) *works in the field* (2394—392 different words); and (3) *works outside the field* (1802—417 different words). Table 1 presents a comparative description of the macrostructural indicators and of the connections between words, which characterize the semantic networks of the three groups of undergraduate students.

**Table 1:** Macrostructural data of the giant component of critical network

Networks	*n*	*m*	*〈k〉*	*Δ*	*D*	*L*
Does not work	233	327	2.81	.012	27	9.75
Works in the field	169	228	2.70	.016	27	10.10
Works outside the field	278	416	2.99	.011	15	6.58

*Notes*: *n* number of vertices, *m* number of edges, *〈k〉* average degree, *Δ* density, *D* diameter, *L* average shortest path

The data indicate that those who *do not work* and who *work in the field* have more consonant patterns compared to the *works outside the field* group. For instance, Table 1 presents that the average shortest path (*L*) of these networks and the greatest possible distances between words (*D*) are similar between students who *do not work* and who *work in the field* when compared to the network of students who *work outside the field*. In other words, the larger diameters found in the *does not work* and *works in the field* networks represent the diversity of existing paths among the words, especially the paths that connect subsets. Moreover, many of these paths took the form of strong links between the words of different subgroups.

The comparison of the data in Table 1 indicates that there was greater heterogeneity of words in the critical network of who works outside the field group (278 vertices), signaling a greater dispersion in the establishment of a sense of professional identity. Conversely, who works in the field presents a more concise set of words to express professional identity (169 vertices). Who does not work is situated between these two (233 vertices). Likewise, the indicator related to the number of edges indicates that work in the field establishes a smaller number of links between words, precisely because it is a more concise network of meanings to express professional identity (228 edges). The works outside the field group have more words in its network and, therefore, also a greater number of edges (416), confirming the characteristic of being a more dispersed and heterogeneous network to express the sense of professional identity. Again, the does not work group is situated between the other two groups (327 edges).

The average degree of connection between the words was similar between the three groups but maintains the pattern of greater dispersion for the who works outside the field group (2.99) and less dispersion for the who works in the field group (2.70). This indicates that the group of university students who have experience in the professional field during higher education can express the meanings of professional identity more internally. The works outside the field group, on the other hand, have a smaller indicator of connection between words, characterizing a more diffuse sense for professional identity. Those who do not work yet occupy an intermediate position (2.81). The results already mentioned are confirmed by the density of the network—greater for students who work in the field (.016)—indicating a group with a greater connection in terms of the meanings of professional identity, with the works outside the field group being the one in which connections are made more tenuous (.011).

Finally, we observed that the diameter of the who does not work network and the who works in the field network is the same (27), but very different from the diameter of the who works outside the field network (15). These results indicate that a greater distance between the words in the who works outside the field network, that is, the meanings attributed to professional identity by this group of university students tend to be less concise, with greater distance between words. The data relating to the average shortest path between the words ratifies the previous results, insofar as it points to a similar pattern between the does not work network and the works in the field network (9.75 and 10.10, respectively), which present an average shortest path greater than that of the works outside the field network (6.58). This result indicates greater paths between the words related to professional identity among university students who have work experience in the field and those who dedicate themselves exclusively to undergraduate studies, without previous work experiences. On the one hand, both groups build more complex networks of meaning for professional identity. On the other hand, college students who work outside the field demonstrate a more restricted process of elaboration about the phenomenon, in which the connection between words occurs from shorter paths.

**Fig. 2:**
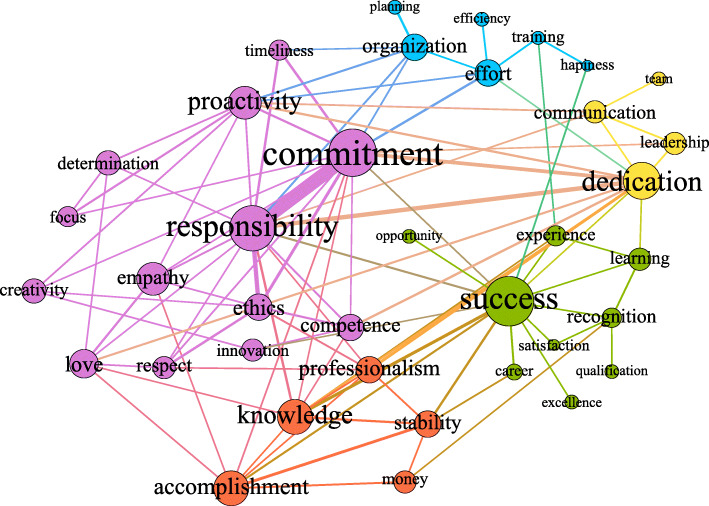
Semantic network of professional identity for the group of undergraduate students who do not work. *Notes.* The colors of the connections between the words represent the communities; the size of the nodes is proportional to their centralities of intermediation, and the thickness of the edges is proportional to the value of the IF index

**Fig. 3:**
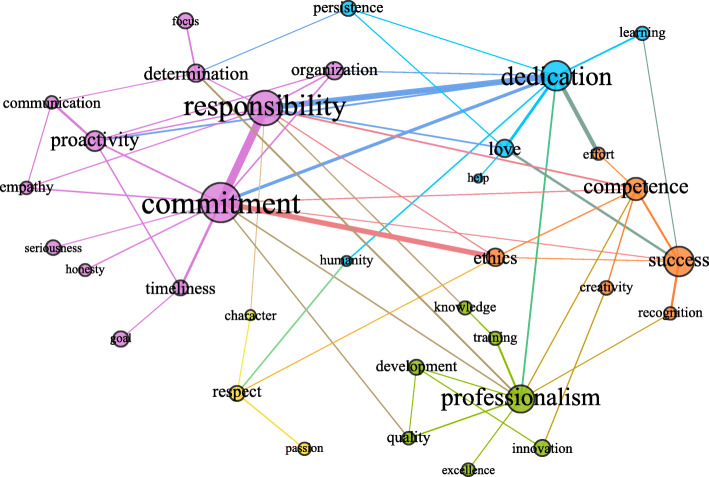
Semantic network of professional identity for the group of undergraduate students who work in the field*. Notes*. The colors of the connections between the words represent the communities; the size of the nodes is proportional to their centralities of intermediation, and the thickness of the edges is proportional to the value of the IF index

**Fig. 4:**
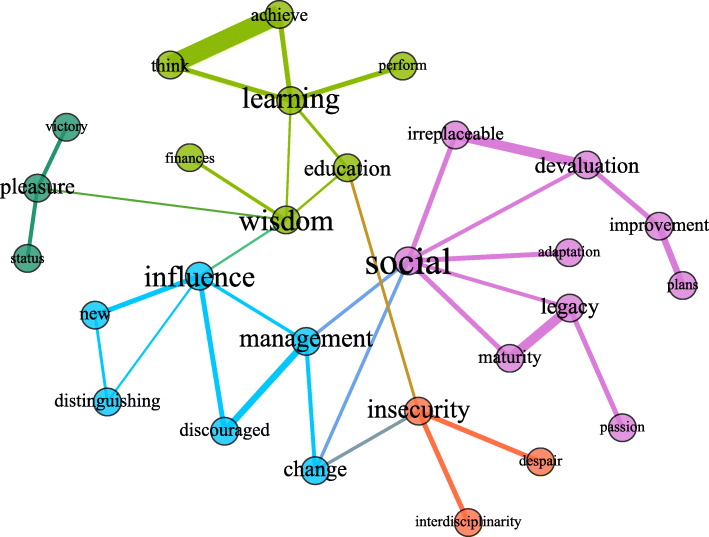
Semantic network of professional identity for the group of undergraduate students working outside their field. *Notes*. The colors of the connections between the words represent the communities; the size of the nodes is proportional to their centralities of intermediation, and the thickness of the edges is proportional to the value of the IF index

Figures 2, 3, and 4 presented in each of the subgroups, in turn, represent the giant component of their respective critical networks, that is, the largest subset of densely connected words. In them, the circles are the words (vertices), and the edges (lines) represent the connections between them. The size of the circles is proportional to the size of the centrality of intermediation of that word in the network, indicating the proportion in which a passage is necessary for other words to connect and acquire meaning in the context of the utterances. As for the thickness of the edges, it varies depending on the IF of the connected word pair. Thus, the thicker it is, the greater the strength of the relationship between them.

The colors represent the communities (the subsets of words that are more cohesive) when their connections are greater than the connections they have with the other vertices in the network. Pink represents the largest community, followed by green, blue, orange, and yellow. Colors different from these represent words belonging to weak communities, in which the words are poorly connected in relation to the words outside the community. The next topics present the figures and the summaries of the macrostructural data of the three networks.

### Professional identity for those who do not work

The critical network of the words uttered by the undergraduate students who do not work contains 233 words, with 327 edges between them. Figure 2 shows the giant component of this network, with 37 words and 99 connections between them, resulting in a density of 15% (the highest among the three analyzed networks). The words *success*, *commitment*, *dedication*, and *responsibility* are the main paths of connection due to their high degree of intermediation in the network. Of these four words, the strength of the relationship between the words in the *commitment* and *responsibility* pair, and between this pair and the word *dedication* draws attention. This means to say that a representative contingent of students uttered these word pairs: *commitment* and *responsibility*, *commitment* and *dedication*, and *responsibility* and *dedication*. The IF values suggest that these pairs were uttered in a very associative and faithful manner. For example, in addition to many individuals uttering the word *commitment*, most also uttered the word *responsibility*.

Although the word *success* has no strong connections, it has the highest degree of intermediation, which expands its role as an axis of meaning as it is the main path for access to the other words in the network. The link of the word *success* with the other words in the network has, in general, a weaker IF, but is associated with a high centrality degree, indicating that it did not have more faithful or predominant word pairs, but formed many pairs with several different words. From the moment that success forms weak links and occupies high intermediation, it takes on the status of a structural hole in the network, and its absence would cause a high fragmentation in the general structure of the network (Burt, 2002). This reinforces the importance of success as a central axis of meaning for the professional identity of the group that does not work.

Despite the relevance of the words *commitment*, *dedication*, and *responsibility*, and the strong relationship between them, other words stand out in this network, whether due to high intermediation and/or the degree centrality, with highlight to *effort*, *recognition*, *organization*, *communication*, *accomplishment*, *proactivity*, *knowledge*, *stability*, *professionalism*, *competence*, *love*, *ethics*, and *empathy*. Some words are exclusive to the giant component of the group network that does not work, namely, *career*, *planning*, *experience*, *opportunity*, *money*, *leadership*, *team*, *efficiency*, *accomplishment*, *satisfaction*, and *happiness*.

### Professional identity for those who work in the field

The network of words the students who work in their field of training uttered contains 169 words, with 228 edges between them. Figure 3 shows the giant component, composed of 34 words and 65 connections between them, which accounts for 12% of all possible relationships (density = 12%). The words *commitment*, *responsibility*, *professionalism*, and *dedication* are the essential paths of connection between the other words in the network. The strength of the relationship, obtained from the IF of the following pairs, draws attention: *commitment* and *responsibility*, *responsibility* and *dedication*, and *commitment* and *ethics*. This means to say that many of the students who have a paid activity in the same field of training uttered these words together.

It is worth noting that the word *ethics*, in addition to having a strong relationship with *commitment*, is the shortest path between *commitment* and *success*. The word love is one of the few shorter and stronger paths between *responsibility* and *success*. What sets it apart from other paths, such as the word competence, is the fact that it belongs to a niche group of meaning that is different from the niches in which *responsibility* and *success* are found. Some words are exclusive to the giant component of the network of the group that works in the field, namely, * help*, *humanity*, *character*, *honesty*, *goal*, *development*, *persistence*, and *seriousness*.

### Professional identity for those who work outside the field

The critical network of words the students working outside their field of training uttered contains 278 words, with 416 edges between them. As part of a critical network with few connections, the largest community, in the network, of those working outside their field (Fig. 4) is made up of 28 words and 35 connections between them, resulting in a 9% density (the lowest density among the three networks). The pattern of less frequent connections is also expressed in the relationships between different niches of meaning. Furthermore, these connections between subgroups are weak (expressed in the thinner thickness of the lines), when compared to the intensity of the intragroup links.

The yellow group, composed of the words *pleasure*, *status*, and *victory*, has only a single and weak connection with the rest of the network through the word *wisdom*. The words with greater centrality of intermediation and degree for this component are *social*, *influence*, and *wisdom*. Excluding these words would fragment the network into at least four parts. The word pairs with stronger links are * think* and * achieve*; *irreplaceable* and *devaluation*; and, finally, *legacy* and *maturity*.

Almost all words in the giant component of the network of those working outside their field are exclusive to this group, except for *learning* and *passion*. Many of these words, in addition to being exclusive, denote meanings not expressed earlier. At first, the presence of negative words draws attention: *discouraged*, *despair*, *devaluation*, and *insecurity*. Observing some of the triads these words compose in the context of utterances by undergraduate students working outside the field calls for reflections, namely, (*i*) *change-social-insecurity*, (*ii*) *management-change-social*, and (*iii*) *discouraged-influence-management*. Also, we observed direct links between the word insecurity and the words *interdisciplinarity*, *despair*, *change*, and *education*.

## Discussion

The objective of this study was to assess, by analyzing semantic networks, the role of work experiences in the meanings that undergraduate students attribute to professional identity. The analysis of the three networks evidences that work experiences are related to the construction of the undergraduate students’ meaning about professional identity. Thus, having no work experience, having experience in the field of the course, and having experience outside the field of training results in different semantic networks.

Taking the three networks together, the evidence expressed by the measures and the comparison of these networks highlight the works outside the field network, which is the only one with a negative connotation. The semantic networks of those who *do not work* and those who *work in the field* have a similar macrostructure and many coincident words. But the analysis of the connection flow between these words, along with other characteristics such as words exclusive to each network, allows identifying important distinctions between the networks of students who *do not work* and who *work in the field.*

If only the main components are observed, the density of the does not work network is higher compared to that of the *works in the field* network. This is the result of a considerable proportion of words with a high power of intermediation associated with a high connection between subgroups of niches of meaning. Though with fewer connections, the *works in the field* network contains more word pairs with high IF. Comparatively, the utterances of undergraduate students who *work in the field* tend to evidence greater clarity as to the central elements involved in the process of professional identity construction.

Despite several coincident words, the meanings of professional identity differ in these networks. The word *success* appears less in the *works in the field* network and is directly related to the words *ethics* and *love*. The first is the main path to *commitment*, and the second is the path straight to *responsibility*. In the network of undergraduate students who do not work, the words *love* and *ethics* depend on the link between other words for success to be achieved.

The comparison between the three networks can also be based on the exclusive words that appear in each one. Thus, the semantic network of the *does not work* group presents a set of exclusive words concerning professional identity, closely associated with the common sense of what professional success would be, namely, *career*, *planning*, *experience*, *opportunity*, *money*, *leadership*, *team*, *efficiency*, *accomplishment*, *satisfaction*, and *happiness*. This result confirms a romanticized view of the profession among undergraduate students who enter the college world, but who still do not know the job market well (Gondim et al., 2016). These words also refer to a yearning for personal fulfillment through the exercise of the profession, as it is noteworthy that words such as accomplishment, satisfaction, and happiness only appeared in the critical network of this group. Besides, in the *does not work* group, some words refer to a greater degree of concreteness, such as *planning*, *career*, *opportunity*, *leadership*, *team*, and *money*.

The undergraduate students who *work in the field* also presented exclusive words in their critical network about professional identity that are very distant from the words of those who do not work, which were * help*, *humanity*, *character*, *honesty*, *goal*, *development*, *persistence*, and *seriousness*. They are not only related to values, but also to a concern about the social impact of their professional practice. In this sense, working in the field comes close to Super’s view of career (Super, 1980), as it reinforces the objective and subjective elements present in career development. These findings confirm the studies that point to the effects of practical experiences beyond the HEIs, especially when related to one’s field of training (Hunter et al., 2007; Mourão et al., 2020).

The critical network of the undergraduate students who *work outside the field* is formed, almost entirely, by exclusive words, that is, words that do not appear in the critical networks of the other groups of undergraduate students. This suggests an attribution of meaning to this group’s very peculiar professional identity. While the group that *does not work* shows a romanticized view of professional practice, and the group that w*orks in the field* expresses a network of meanings associated with the values and social impact of their profession, those who *work outside the field* utter very different words that unveil some suffering in reconciling work and studies, at the same time that there is an expectation for change, whether in terms of social status or learning.

It is as though higher education courses were a bridge capable of leading them from a current work situation for subsistence to another condition in which working is endowed with a purpose. Thus, the exclusive words of this network are *adaptation*, * achieve*, *despair*, *devaluation*, *distinguishing*, *education*, * perform*, *finances*, *management*, *influence*, *insecurity*, *irreplaceable*, *interdisciplinarity*, *legacy*, *maturity*, *improvement*, *change*, *new*, * think*, *pleasure*, * project*, *wisdom*, *social*, *status*, *and victory*.

On the one hand, work experiences, even those outside one’s field of training, seem to contribute to the construction of meanings for a professional identity that reflects the results of experiential learning discussed by Kolb (1984), since the critical network of this group was the only one that presented the words *adaptation*, *to achieve*, *to perform*, *improvement*, *change*, * think*, and * project*. On the other hand, we observed direct links between the word *insecurity* and the words *interdisciplinarity*, *despair*, *change*, *and education*, which reinforces the idea that negative feelings can reflect a tension between one’s field of training and field of job insertion. There is, therefore, a tension between identification with a certain group that shares the same theoretical and practical domain (collegemates) and opposition to or distancing from other professional groups (co-workers). Such tension in this group of undergraduate students may express yet another division between the field students chose to study and the field in which they work. That could possibly cause some implications as to doubts about whether or not they should stay in their current job and the decisions necessary for structuring a career.

Therefore, broadly speaking, we observed a greater similarity between the critical network of the undergraduate students who do not work and those who work in the field, than between these networks and the network of those who work outside their field. This result points to the fact that, even though experiential learning occurs in the work experiences of undergraduate students who work, the processes of professional-identity construction evidence that working outside the field generates tensions. These tensions may be associated with the fact that working outside the field is not necessarily a decision aimed at acquiring work experiences, with the decision being oftentimes associated with a need for survival, with possible conflicts for future career decisions. The understanding of these tensions may be associated with the very concept of professional identity, which refers to the perception of inclusion in a certain social group that shares a specific domain of technical and work-related knowledge (Tajfel, 1983). In this sense, undergraduate students working outside their field are inserted in groups with different knowledge domains, which may cause tensions.

## Conclusions

In conclusion, it is worth pondering that professional identity is under construction throughout one’s university training, being constituted from the interaction between the individual’s skills and social learning, with highlight to those derived from possible work experiences. Therefore, the critical networks of the groups with different work experiences point to different meanings attributed to professional identity, even though all are in the university training process.

This study contributes to the field of studies on professional identity, in both theoretical and methodological scopes. At the theoretical level, we observed that the central meanings associated with the professional identity of undergraduate students tend to favor competencies and values that are more comprehensive and transversal to the professions than specific competencies of a given professional category. We found that the work experiences lived or not during the undergraduate course contribute to the construction of professional identity, with a production of different meanings among university students who *do not work*, those who *work in the field*, and those students who work in different activities than their area of study. The research reveals that when the students experience the role of workers beyond their area of training, they use more words of negative content when reflecting on their professional identity. The opposite happens when they experience the role of workers in their area of study.

In the methodological scope, the study innovates by privileging the critical network as the central axis of meaning of semantic networks. This strategy proved to be fruitful, especially when combined with theories that favor narrative as an axis for understanding psychosocial phenomena. Thus, the use of the critical network analysis method allowed us to identify the main axis of meaning attributed to professional identity.

The results obtained in this research have implications for these students’ professional development as the meanings the three groups surveyed attributed to professional identity are quite different. Also, these work experiences likely entail implications for the career trajectories of these university students. These results can support the decisions of academic managers, either in terms of the curriculum proposal for undergraduate courses, or in the implementation of differentiated pedagogical strategies according to the work experiences of university students.

Thus, the main advances that this research has brought to the area refer to the deepening of the debate on the advantages and disadvantages of working concurrently with training, with emphasis on the tensions generated when work is not in line with the training area. Despite these contributions, the research carried out has some limitations referring to the convenience sample and the cross-sectional design.

A future research agenda could include longitudinal studies that assess changes in the meanings attributed to professional identity over time, allowing the observation of how this narrative is constructed, and the events in it that influence the daily lives of undergraduate students. Furthermore, studies that assess patterns of meaning attributed to professional identity by different working groups can contribute in epistemological conceptual terms to the field of studies on professional identity. Finally, we suggest studies that consider different working groups and check differences between the area of studies and those groups.

## Data Availability

Data are available from the first author.
